# History of Disseminated Intravascular Coagulation (DIC) Research and My Personal Research History

**DOI:** 10.14789/ejmj.JMJ25-0011-P

**Published:** 2025-06-20

**Authors:** TOSHIAKI IBA

**Affiliations:** 1 Faculty of Medical Science, Juntendo University, Chiba, Japan; Faculty of Medical Science, Juntendo University Chiba, Japan

**Keywords:** disseminated intravascular coagulation, diagnostic criteria, coagulation, fibrinolysis, sepsis

## Abstract

Disseminated intravascular coagulation (DIC) is like a cloud. We really can see it, but we never feel or touch it. This means that we can diagnose it, but we still do not know how that happens and how to manage it. DIC was first recognized as an uncontrollable bleeding disorder complicated by critical diseases. Curiously, despite such bleeding tendency, pathological examination revealed systemic micro-clot formation in multiple organs. Soon after, these specific findings were revealed as the result of excess coagulation followed by consumptive coagulopathy. Numerous factors, such as imbalanced coagulation/fibrinolysis, endothelial damage, platelet activation, and intravascular inflammation, are involved in the pathogenesis. However, the whole story has not been clarified yet, and DIC is also recognized as "Disseminated Intracerebral Confusion." In nearly 40 years, several diagnostic criteria were proposed but never integrated. As for the sepsis-associated DIC, the International Society on Thrombosis and Haemostasis recommended a two-step approach by Sepsis-Induced Coagulopathy (SIC) followed by overt DIC criteria. In this approach, the early (compensated)-phase is diagnosed by SIC, and the late (decompensated)-phase is diagnosed by overt DIC criteria. Regarding treatment, we are very sorry, but only the treatment for underlying conditions is recommended. However, aggressive anticoagulant therapies for early-phase DIC have been explored in Japan. We expect the effects of anticoagulant therapy to be proven in the near future.

The research on disseminated intravascular coagulation (DIC) has been a significant part of my life. However, since we still cannot fully answer “What is DIC?”, we look back at the history of DIC and attempt to anticipate future insights. DIC was first recognized in the late 19th to early 20th centuries as a coagulation disorder associated with sepsis and obstetric complications. At the time, physicians struggled to understand a unique condition, in which the primary symptom was bleeding, but the histology showed systemic clot formation. But soon after, it was referred to as “consumptive coagulopathy,” as it was characterized by excessive clot formation leading to the depletion of coagulation factors and platelets^1)^.

In the 1950s, researchers began to understand that DIC was not merely a bleeding disorder but rather a complex process involving widespread microvascular thrombosis followed by unbalanced fibrinolysis. The term “DIC” was established, and it was recognized as a secondary condition triggered by various underlying diseases, including sepsis, malignancy, trauma, obstetric disorders, and venom toxin.

By the 1970s, DIC diagnosis had mainly been made based on clinical symptoms. However, that was not objective, and it was often too late to do anything for the patients. Therefore, laboratory tests such as platelet count, prothrombin time (PT), fibrinogen levels, and fibrin-related markers, i.e., fibrin/fibrinogen degradation products (FDPs), and D-dimer were proposed as key markers for DIC diagnosis. In 1988, the Japanese Ministry of Health and Welfare introduced diagnostic criteria (JMHW- DIC) comprised of the above four items. Following this, the International Society on Thrombosis and Haemostasis (ISTH) released a similar framework for DIC^2)^. The key advantage of the ISTH-DIC criteria is their recognition of the early phase of DIC as non-overt DIC, acknowledging that DIC is not solely a severely decompensated coagulation disorder but also includes a compensated phase (Figure 1).

**Figure 1:**
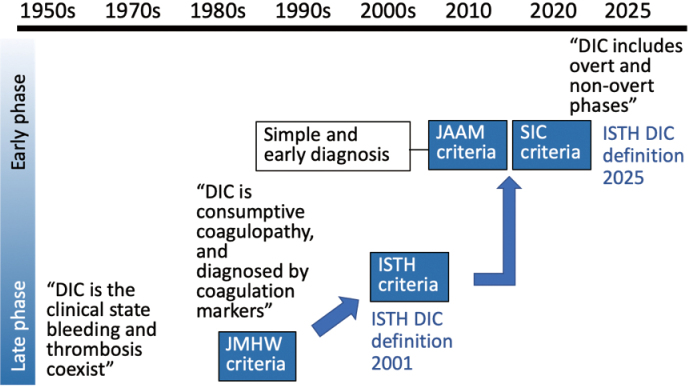
Historic changes in diagnostic criteria for DIC The first diagnostic criteria for disseminated intravascular coagulation (DIC) was established in Japan by the Japanese Ministry of Health and Welfare (JMHW) in 1988. Following this, The International Society on Thrombosis and Haemostasis (ISTH) released similar criteria comprised of platelet count, fibrin-related markers, prothrombin time, and fibrinogen. The Japanese Association on Acute Medicine (JAAM) and ISTH released either JAAM DIC criteria or Sepsis-induced Coagulopathy (SIC), which are both simple and easy to use. In 2025, the ISTH announced that DIC includes early-phase DIC.

I graduated from Juntendo University in 1984 and began my research around 1989 in the United States, coinciding with the launch of JMHW-DIC criteria. I still vividly recall the profound impact of this event, as it marked the beginning of a continuous stream of studies emerging from Japan. During the 2000s, treatment strategies based on DIC pathophysiology began to emerge. Anticoagulants such as recombinant activated protein C (rAPC)^3)^, antithrombin^4)^, recombinant tissue factor pathway inhibitor^5)^, and recombinant thrombomodulin (rTM)^6)^ were explored. However, since most of the randomized controlled studies (RCTs) were performed in patients with severe sepsis and not in sepsis-associated DIC, the effect was proven only in the RCT examined the effect of rAPC (PROWESS). Regrettably, rAPC was withdrawn from the market after the repeated failures of the clinical trials. One of the main reasons most anticoagulant RCTs failed to show positive results was the inclusion of inappropriate patient populations. In contrast, the phase 3 study of rTM targeted DIC and was approved following clinical trials in 2008. Since then, rTM has since been widely used in Japan for the treatment of DIC. Although a recent RCT (SCARLET) could not show a beneficial effect, rTM is marking a significant milestone in DIC management^7)^.

Another reason for the failure was the delay in treatment initiation. As the young investigators of the Japanese Association for Acute Medicine (JAAM), we argued that the diagnoses made using the JMHW and overt DIC criteria were too late to effectively start anticoagulant therapy. We built the JAAM DIC criteria and launched it in 2006 to enable earlier diagnosis and intervention^8)^. I still remember clearly the day I urged the head of the JAAM board to establish criteria that would enable early treatment.

The other reason is the heterogenicity of DIC. Since the early 2000s, researchers have recognized that DIC is not a single condition but rather a spectrum of disorders that differ based on the underlying disease^9)^. This led to the classification of DIC into different subtypes, including suppressed-fibrinolysis type DIC (common in sepsis), enhanced-fibrinolysis type DIC (common in acute promyelocytic leukemia and early phase trauma), and balanced- fibrinolysis type DIC (common in solid tumors)^10)^. It is important to know that anticoagulants should be selected properly depending on the underlying diseases and coagulation/fibrinolytic balance.

Despite the advancement of knowledge and clinical experiences, the prevalence of DIC diagnosis has not become popular outside Japan. This is mainly because the criteria are complex, and there is a lack of effective treatment. As the chairperson of the ISTH Scientific Standardization Committee, I have established and introduced the concept of Sepsis-Induced Coagulopathy (SIC) and its diagnostic criteria in 2019, paving the way for the development of sepsis-specific DIC diagnostic criteria^11, 12)^. SIC classifies the early phase of DIC associated with sepsis, enabling the identification of appropriate patients for anticoagulant therapy.

Following this, we updated the definition of DIC in 2025. DIC is now defined as: “An acquired, life- threatening intravascular disorder characterized by systemic coagulation activation, dysregulated fibrinolysis, and endothelial injury, resulting in microthrombosis. DIC arises from various underlying etiologies and progresses from a potentially asymptomatic early phase to an advanced phase with hemorrhage and/or organ dysfunction^13)^.” In accordance with this more comprehensive definition, we propose to establish more tailored diagnostic criteria that detect early-phase DIC based on the underlying disease.

The history of DIC research is a testament to the ongoing advancements in thrombosis and hemostasis research, highlighting the interplay between fundamental discoveries and clinical practice. Nowadays, SIC and ISTH diagnostic criteria have become widely used, emphasizing the importance of early diagnosis and intervention. Future research aims to develop personalized treatment strategies, incorporating genomic studies, biomarker-based diagnosis, and artificial intelligence (AI)-assisted risk assessment. As our understanding of DIC continues to evolve, new therapeutic approaches will likely improve patient outcomes in various critical care settings.

## Author contributions

TI wrote and reviewed the manuscript. He read and approved the final manuscript.

## Conflicts of interest statement

The author declares that he has no conflict of interest. One author, a member of the JMJ editorial board, was not involved in the peer review or decision-making process for this paper.
